# Effect of *HXT*1 and *HXT*7 hexose transporter overexpression on wild-type and lactic acid producing *Saccharomyces cerevisiae *cells

**DOI:** 10.1186/1475-2859-9-15

**Published:** 2010-03-09

**Authors:** Giorgia Rossi, Michael Sauer, Danilo Porro, Paola Branduardi

**Affiliations:** 1 Università degli Studi di Milano-Bicocca, Department of Biotechnology and Bioscience, P.zza della Scienza 2, 20126 Milano, Italy; 2 School of Bioengineering, FH Campus Wien, University of Applied Sciences, Muthgasse 18, 1190 Vienna, Austria; 3 Department of Biotechnology, BOKU, University of Natural Resources and Applied Life Sciences, Muthgasse 18, 1190 Vienna, Austria

## Abstract

**Background:**

Since about three decades, *Saccharomyces cerevisiae *can be engineered to efficiently produce proteins and metabolites. Even recognizing that in baker's yeast one determining step for the glucose consumption rate is the sugar uptake, this fact has never been conceived to improve the metabolite(s) productivity.

In this work we compared the ethanol and/or the lactic acid production from wild type and metabolically engineered *S. cerevisiae *cells expressing an additional copy of one hexose transporter.

**Results:**

Different *S. cerevisiae *strains (wild type and metabolically engineered for lactic acid production) were transformed with the *HXT*1 or the *HXT*7 gene encoding for hexose transporters.

Data obtained suggest that the overexpression of an Hxt transporter may lead to an increase in glucose uptake that could result in an increased ethanol and/or lactic acid productivities. As a consequence of the increased productivity and of the reduced process timing, a higher production was measured.

**Conclusion:**

Metabolic pathway manipulation for improving the properties and the productivity of microorganisms is a well established concept. A high production relies on a multi-factorial system. We showed that by modulating the first step of the pathway leading to lactic acid accumulation an improvement of about 15% in lactic acid production can be obtained in a yeast strain already developed for industrial application.

## Background

Natural *Saccharomyces cerevisiae *cells have long been utilized as very efficient biocatalysts, thanks to their native enzymatic capabilities. Ethanol, single cell proteins, flavours and fragrances are among the most traditional examples.

Since about three decades budding yeast can also be engineered and has been used to efficiently produce simple as well as complex molecules. Prominent examples are proteins with pharmaceutical applications, industrial enzymes, organic acids, new bio-fuels, biopolymers, vitamins and steroids, in a single fermentation step [1-8].

Glucose, either derived from starch and/or cellulosic materials, is the main carbon and energy source today available.

An economically sustainable bioprocess leading to the production of a homologous or heterologous low molecular compound requires a high yield (grams of product obtained by gram of substrate), high production titer (g/L) and high productivity (g/L/h) values.

It has been shown that high yields and high production titres can be obtained by recombinant redirection of the carbon flow towards the desired compound. In this respect, prominent examples are the production of lactic-, pyruvic- and malic- acid, glycerol and resveratrol [9-13]. Theoretically, high productivities could be obtained by increasing the carbon consumption rates itself (*i.e*., essentially the glycolytic flux rate). It should be also underlined that an increased productivity, and therefore a reduction of the process duration, is not only implying a reduction in terms of costs. Cell factory viability (and therefore production) has also to be taken into account: very often the production process involves a stressful environment, leading to cell death during fermentation, as in the case of ethanol production, gradually reducing cell viability and thereby biocatalyst concentration [14].

Glucose transport, hexose phosphorylation, phosphofructokinase and pyruvate kinase activities have all been proposed to play central roles in the control of glycolysis flux rates [15-20].

Individual or simultaneous overproduction of glycolytic enzymes resulted either in no increases in glycolytic flux or in only incremental increases [21-25]. Furthermore, attempts to correlate glycolytic flux with enzyme levels under different physiological conditions have generally failed [26-28]. This is likely because the control of glucose catabolism is distributed over several different metabolic controls; in this context, glucose transport has been suggested to be one of the most important players [29,30].

Glucose transport in *S. cerevisiae *relies on a multi-factorial uptake system. More in details, the uptake of glucose by *S. cerevisiae *is controlled by multiple hexose transporters (Hxts) [31]. At least 20 *HXT *genes encoding these transporters have been identified [31,32]. Many and different studies were done to determine the respective biochemical features of these transporters (affinity and capacity), as well as to construct strains deleted in one or more *HXT *genes and to construct chimera proteins combining affinity and capacity of different transporters [33-35]. Remarkably, Otterstedt *et al.*[33] showed that a simple manipulation of the glucose uptake can strongly alter the mode of metabolic control.

Essentially, the various hexose transporters differ considerably in substrate specificity and affinity. Hxt1 and Hxt3 are low-affinity transporters (Km for glucose, ~50 to 100 mM), Hxt4 is a moderately low-affinity transporter, and Hxt2, Hxt6, and Hxt7 are high-affinity transporters (Km for glucose, ~1 to 4 mM) [36,37]. Hxt5 has been shown to be a transporter with intermediate to high affinity [38,39]. Both high- and low-affinity carriers have been shown to have a higher affinity for glucose than for fructose [37]. Analyses of the effect of *HXT *gene inactivation have shown that the hexose carriers Hxt1 to Hxt7 are the main transporters [37]. In this respect, it has been already shown that the ethanol (and CO_2_) productivity and yield (grams of ethanol produced per gram of glucose consumed) can be improved by overexpression of *HXT*1 transporter in *S. cerevisiae *[40-43].

*S. cerevisiae *has long been studied for the production of organic acids like lactic, ascorbic, pyruvic and malic [[4,8,11] and [44]]. Indeed, yeast can grow and survive at low pH values, avoiding the accumulation of the respective salts [4,11].

Here we show improved lactic acid productivities, induced by an increase of the glucose consumption rate. Hxt1 and Hxt7 have been selected for this study. In spite of their different biochemical properties, the overexpression of *HXT*1 or *HXT*7 genes does lead to very similar results in the tested conditions. Moreover, we demonstrate that the increase of the glucose consumption rate has a positive effect not only in respect to microbial productivity and metabolite production, but also on biomass accumulation. Said phenomenon is more or less evident in respect to the yeast background.

## Results

### Effect of *HXT1 *and *HXT7 *overexpression in naturally ethanol producing yeasts

First the effect of the overexpression of the two different hexose transporters in two different yeast genetic backgrounds was studied. The strains GRF18U (the model yeast strain used in our laboratory) [45] and CEN.PK (a generally accepted reference yeast strain) [46] were both transformed with the integrative plasmids p022HXT1 or p022HXT7, respectively carrying the *HXT*1 [47] and *HXT*7 [31] genes under the control of the glycolytic *Sc*TPI promoter (Table 1: for each transformation, at least three independent transformants were analysed in three independent experiments). *HXT*1 and *HXT*7 encode for the two hexose transporters having the lowest and the highest affinity for glucose, respectively.

**Table 1:** Transformed strains used in this study

Strains	Genotype	Plasmid*
**GRF18U**	*MATa, ura3, his3, leu2*	**p022 **(*Sc*TPI; -; *HIS*3)
**GRF18U [*HXT*1]**	*MATa, ura3, his3, leu2*	**p022HXT1 **(*Sc*TPI; *HXT*1; *HIS*3)
**GRF18U [*HXT*7]**	*MATa, ura3, his3, leu2*	**p022HXT7 **(*Sc*TPI; *HXT*7; *HIS*3)
**CEN.PK**	*MATa, ura3-52, his3-11, leu2-3/112, TRP1, MAL2-8c, SUC2*	**p022 **(*Sc*TPI; -; *HIS*3)
**CEN.PK [*HXT*1]**	*MATa, ura3-52, his3-11, leu2-3/112, TRP1, MAL2-8c, SUC2*	**p022HXT1 **(*Sc*TPI; *HXT*1; *HIS*3)
**CEN.PK [*HXT*7]**	*MATa, ura3-52, his3-11, leu2-3/112, TRP1, MAL2-8c, SUC2*	**p022HXT7 **(*Sc*TPI; *HXT*7; *HIS*3)
**CEN.PK [LDH]**	*MATa, ura3-52, his3-11, TRP1, MAL2-8c, SUC2*	**p022 **(*Sc*TPI; -; *HIS*3) **p212LDH **(*Sc*TPI; LDH; *LEU*2)
**CEN.PK [*HXT*1] [LDH]**	*MATa, ura3-52, his3-11, TRP1, MAL2-8c, SUC2*	**p022HXT1 **(*Sc*TPI; *HXT*1; *HIS*3) **p212LDH **(*Sc*TPI; LDH; *LEU*2)
**CEN.PK [*HXT*7] [LDH]**	*MATa, ura3-52, his3-11, TRP1, MAL2-8c, SUC2*	**p022HXT7 **(*Sc*TPI; *HXT*7; *HIS*3) **p212LDH **(*Sc*TPI; LDH; *LEU*2)
**CEN.PKm850 [LDH]**[50]	*MATa, pdc1*::*loxP, pdc5*::*loxP, pdc6*::*loxP, ura3*-*52*	**YEpLpLDH**[50] (*Sc*TPI; LDH; *URA*3) **p022KMX4 **(*Sc*TPI; -; G418^R^)
**CEN.PKm850 [*HXT*1] [LDH]**	*MATa pdc1*::*loxP pdc5*::*loxP pdc6*::*loxP ura3*-*52*	**YEpLpLDH**[50] (*Sc*TPI; LDH; *URA*3) **p022KMX4HXT1 **(*Sc*TPI; *HXT*1; G418^R^)
**CEN.PKm850 [*HXT*7] [LDH]**	*MATa pdc1*::*loxP pdc5*::*loxP pdc6*::*loxP ura3*-*52*	**YEpLpLDH**[50] (*Sc*TPI; LDH; *URA*3) **p022KMX4HXT7 **(*Sc*TPI; *HXT*7; G418^R^)

* Plasmid name. In brackets are reported the promoter, the harboured gene and the selection marker, respectively.

**Figure 1:**
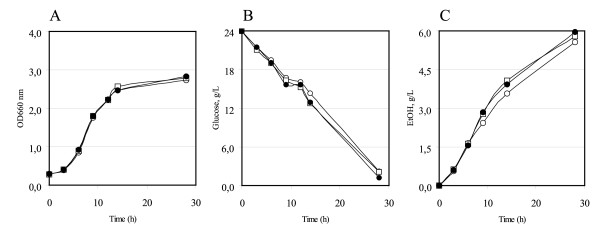
**Overexpression of *HXT*1 or *HXT*7 genes in the *S. cerevisiae *GRF18U strain**. Strains were flask-batch grown in minimal (YNB) medium, with glucose as a carbon source. (A) Growth was measured as optical density (OD 660 nm). (B) Residual glucose, g/L. (C) Ethanol produced, g/L. Data correspond to the mean values of three independent clones independently tested at least three times. Standard error is lower than 0.03%. Open circles: GRF18U-Control. Closed circles: GRF18U [*HXT*1] transformants. Open squares: GRF18U [*HXT*7] transformants.

**Figure 2:**
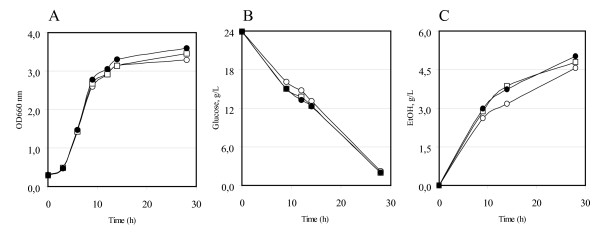
**Overexpression of *HXT*1 or *HXT*7 genes in the *S. cerevisiae *CEN.PK strain**. Strains were flask-batch grown in minimal (YNB) medium, with glucose as a carbon source. (A) Growth was measured as optical density (OD 660 nm). (B) Residual glucose, g/L. (C) Ethanol produced, g/L. Data correspond to the mean values of three independent clones independently tested at least three times. Standard error is lower than 0.03%. Open circles: CEN.PK-Control. Closed circles: CEN.PK [*HXT*1] transformants. Open squares: CEN.PK [*HXT*7] transformants.

Initially, a functional analysis of the cloned *HXT*1 and *HXT*7 genes was performed by examining the effect of their expression in a *hxt1-7 *deleted strain, RE700 [48]. While the control strain grows very poorly on glucose minimal medium, the transformed ones partially resumed the natural ability (data not shown). Then the natural abilities of the control and of the *HXT*1 or *HXT*7 transformed yeasts to produce ethanol were compared. Figure 1 and Figure 2 show the shake-flask batch growth kinetics in defined YNB-2% glucose medium of transformed GRF18U (Figure 1, see also Table 2) and CEN.PK (Figure 2, see also Table 2) yeast strains. Panel (A) shows the growth, measured as optical density (OD_660_), of the wild type strain (open circles), of the *HXT*1 (closed circles) and *HXT*7 (open squares) overexpressing strains. Panels (B) and panels (C) show the glucose consumption and the ethanol production, respectively. The results clearly show that the presence of an additional copy of a hexose transporter leads to a faster glucose consumption rate and a faster ethanol production rate (panels B and C). It can be observed that similar glucose consumption rates have been observed from the different transformed strains, despite of the different biochemical features of the Hxt1 and Hxt7 transporters. A very low glucose concentration should have been tested to evidence the effect of the overexpression of the *HXT*7 transporter (which has the highest affinity for glucose): however, at that concentration the fermentation product formation would have also been very low. The two genes were initially tested because we could not *a priori *predict their positive or, eventually, negative effect.

**Table 2:** Effect of the overexpression of *HXT*1 or *HXT*7 genes in the *S. cerevisiae *GRF18U or CEN.PK strains

Strains		Glc %	YNB%	aa mg/L	Agitation	μ_exp phase [1/h]	Rate Glc Consumption* [g/L/h]	Rate EtOH Production* [g/L/h]	Rate LA Production* [g/L/h]	Y_EtOH** [g EtOH/g Glc]	Y_LA** [g L.A./g Glc]
CEN.PK		2.39	1.34	50	YES	**0.28** +/- 0.05	**-0.77** +/- 0.08	**0.23** +/- 0.09	-	**0.19** +/- 0.11	-
CEN.PK	[*HXT*1]	2.39	1.34	50	YES	**0.29** +/- 0.07	**-0.83** +/- 0.08	**0.27** +/- 0.04	-	**0.21** +/- 0.05	-
CEN.PK	[*HXT*7]	2.39	1.34	50	YES	**0.29** +/- 0.02	**-0.82** +/- 0.08	**0.28** +/- 0.03	-	**0.20** +/- 0.03	-

CEN.PK		5.68	2.68	150	YES	**0.33** +/- 0.05	**-1.66** +/- 0.06	**0.42** +/- 0.01	-	**0.19** +/- 0.00	-
CEN.PK	[*HXT*1]	5.68	2.68	150	YES	**0.33** +/- 0.03	**-1.74** +/- 0.09	**0.44** +/- 0.01	-	**0.20** +/- 0.01	-

GRF18U		2.39	1.34	50	YES	**0.25** +/- 0.05	**-0.69** +/- 0.04	**0.27** +/- 0.05	-	**0.23** +/- 0.01	-
GRF18U	[*HXT*1]	2.39	1.34	50	YES	**0.25** +/- 0.03	**-0.79** +/- 0.08	**0.32** +/- 0.06	-	**0.25** +/- 0.08	-
GRF18U	[*HXT*7]	2.39	1.34	50	YES	**0.25** +/- 0.04	**-0.80** +/- 0.07	**0.31** +/- 0.06	-	**0.24** +/- 0.06	-

CEN.PK	[LDH]	2.04	1.34	50	YES	**0.12** +/- 0.04	**-0.38** +/- 0.07	**0.09** +/- 0.08	**0.08** +/- 0.09	**0.17** +/- 0.01	**0.21** +/- 0.01
CEN.PK	[*HXT*1] [LDH]	2.04	1.34	50	YES	**0.14** +/- 0.08	**-0.40** +/- 0.05	**0.10** +/- 0.04	**0.09** +/- 0.06	**0.19** +/- 0.09	**0.24** +/- 0.01

CEN.PK m850	[LDH]	9.14	1.70	-	YES	**0.02** +/- 0.00	**-1.21** +/- 0.00	-	**0.77** +/- 0.00	-	**0.67** +/- 0.00
CEN.PK m850	[*HXT*1] [LDH]	8.96	1.70	-	YES	**0.03** +/- 0.09	**-1.22** +/- 0.08	-	**0.85** +/- 0.02	-	**0.76** +/- 0.02

CEN.PK		2.13	1.34	150	NO	**0.26** +/- 0.03	**-1.22** +/- 0.03	**0.41** +/- 0.08	-	**0.40** +/- 0.03	-
CEN.PK	[*HXT*1]	2.13	1.34	150	NO	**0.26** +/- 0.04	**-1.30** +/- 0.02	**0.45** +/- 0.02	-	**0.45** +/- 0.08	-

CEN.PK		5.68	2.68	150	NO	**0.23** +/- 0.01	**-4.30** +/- 0.06	**1.37** +/- 0.05	-	**0.31** +/- 0.08	-
CEN.PK	[*HXT*1]	5.68	2.68	150	NO	**0.23** +/- 0.04	**-4.36** +/- 0.07	**1.58** +/- 0.06	-	**0.36** +/- 0.09	-

Table summarizes all the data obtained with wild type and engineered yeasts (see first column) grown in the indicated conditions (see columns 2-5). The specific growth, glucose consumption, ethanol and lactate production rates together with yield for ethanol and lactic acid production are given. For the described determinations, the respective standard error is indicated. **Glc**: glucose. **aa**: aminoacids. **LA**: lactic acid. * determined in exponential phase. ** determined at the timing of the highest metabolite (ethanol or lactic acid) production.

Indeed, similar effects were also obtained when glucose concentration was increased to 5% (Table 2, only data obtained in the CEN.PK background overexpressing the *HXT*1 gene are shown). Biomass production is also increased, particularly in the CEN.PK yeast background. We do not have any explanation for that.

It is also important to underline that during balanced exponential growth, the specific growth rates of the control and the transformed strains are equal (Table 2). A very similar correlation (*i.e*., increased glucose consumption *vs *higher metabolite and higher biomass production) has been previously observed in our laboratory [49]. All the data are summarised in Table 2: the glucose consumption rate (at least in the first phases of the process) and the ethanol productivity and production were improved by overexpressing *HXT*1 or *HXT*7 in the two different yeast genetic backgrounds tested. It needs to be underlined that when cells were grown in medium containing 5% glucose, also the nitrogen content was consequently incremented (see Methods), determining the development of a higher biomass. This justifies the higher glucose consumption and ethanol production rates measured in these growth conditions (Table 2).

Finally, when transformants were grown in the above described media but under micro-anaerobic conditions, similar results have been obtained in respect to growth rate and to substrate consumption and ethanol production (Table 2). Remarkably, in this case the improvement in the ethanol yield of the transformants was higher as the glucose consumption rate (Table 2).

### Effect of *HXT1 *overexpression in yeast engineered for lactic acid production

The CEN.PK *S. cerevisiae *strain harbouring the integrative p022HXT1 expression vector was further transformed with the multicopy plasmid p212LDH bearing an engineered *Lactobacillus plantarum *LDH gene [49] under the control of the glycolytic *Sc*TPI promoter (Table 1). We can briefly comment that very similar results were obtained in GRF18 background and overexpressing the *HXT*7 gene in both yeasts (data not shown).

**Figure 3:**
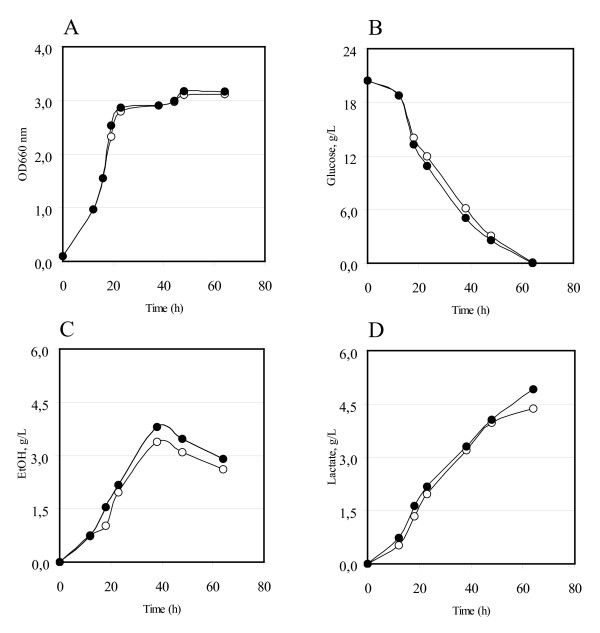
**CEN.PK *S. cerevisiae *strain overexpressing the *HXT*1 gene or the *HXT*1 and the *Lp*LDH gene**. Strains were flask-batch grown in minimal (YNB) medium, with glucose as a carbon source. (A) Growth was measured as optical density (OD 660 nm). (B) Residual glucose, g/L. (C) Ethanol produced, g/L. (D) Lactate produced, g/L. The data correspond to the mean values of three independent clones independently tested at least three times. Standard error is lower than 0.03%. Open circles: CEN.PK [LDH]-Control. Closed circles: CEN.PK [*HXT*1] [LDH] transformants.

Independent transformants were shake flask cultured in YNB-minimal medium. Figure 3 shows the behaviours of the LDH (open circles) and of the *HXT*1-LDH (closed circles) overexpressing strains. Panels (A) and (B) report the cell density (as OD 660 nm) and the glucose consumption, while panels (C) and (D) the ethanol and the lactate accumulation during growth on defined YNB-2% glucose based medium. It is important to underline that while the transformed CEN.PK strains shown in Figure 2 require uracil and leucine for growth, the CEN.PK transformed strains shown in Figure 3 require only leucine. It is reasonable to assume that because of that a slightly different amount on biomass accumulation was observed (compare Figure 2 and Figure 3).

It was observed also in these transformants that an additional copy of *HXT*1 lead to an increase in glucose consumption (Figure 3, panel B). Remarkably, both the ethanol and the lactic acid productivities and titres are improved (Figure 3, panels C and D).

### Effect of *HXT1 *overexpression in homolactic yeasts

**Figure 4:**
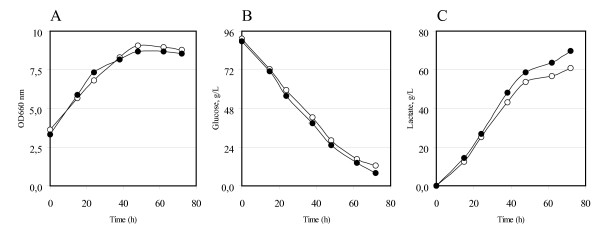
**Lactic acid production in the CEN.PK m850 [LDH] strain overexpressing the *HXT1 *gene**. (A) Growth was measured as optical density (OD 660 nm). (B) Residual glucose, g/L. (C) Lactic acid produced, g/L. The data correspond to the mean values of three independent clones independently tested at least three times. Standard error is lower than 0.03%. Open circles: CEN.PK m850 [LDH]-Control. Closed circles: CEN.PK m850 [*HXT*1] [LDH] transformants.

Considering the improved product titres and productivities obtained, we tested the lactic acid production in the engineered host strain CEN.PK m850 [LDH] (Table 1), *ad hoc *constructed for being a low-pH homolactic producing yeast [50]. Said strain does not produce ethanol because it is totally devoid of pyruvate decarboxylase (Pdc) activity, it bears the *L. plantarum *LDH on a multicopy yeast expression plasmid and finally it has been selected, with an inverse metabolic engineering approach, for its acid tolerance. Figure 4 shows the behavior of the CEN.PK m850 [LDH] strain and of the same strain expressing an additional copy of the *HXT*1 transporter cultivated with 90 g/L of glucose. Independent transformants were tested for glucose consumption and lactic acid production. Under these growth/production conditions both strains did not consume all the glucose present in the medium. The control strain produced approximately 60 g/L of lactic acid at very low pH (after 70 h from the inoculum the external pH value was lower than 3.0). In the strain expressing an additional *HXT*1 copy no improvement in biomass production was observed, while the measured glucose consumption was faster; as a consequence, it could produce a considerable additional amount of lactic acid (about 15% more), or the same amount in a shorter period of time (about 1,4 times faster) (see also Table 2). In line with the previously described kinetics, similar results have been obtained overexpressing the *HXT*7 gene in the CEN.PK m850 background (data not shown).

This last experiment shows the successful application of what was previously shown in laboratory strains (Figure 3) also in a strain already developed and optimised for industrial productions.

In Table 2 the data related to the lactic acid producing m850 strain are also summarised and included.

## Discussion

The hexose transporter gene family in *S. cerevisiae *contains the sugar transporter genes *HXT1 *to *HXT17, GAL2 *and the glucose sensor genes *SNF3 *and *RGT2 *[31-33]. *HXT*1 and *HXT*3 genes have already been overexpressed in yeasts. More in detail, the effect of the overexpression of *HXT1* gene has been tested in a *S. cerevisiae* strain [42] during growth on complex-rich based media. A significant increase on the ethanol productivity (g/L/h) was observed. Also the ethanol yield, expressed as gram of ethanol produced per gram of substrate consumed, showed a significant (3%) improvement. This is in line with our findings. However, Gutiérrez-Lomelì *et al.*[42] observed no significant effects on final ethanol concentration. On the other hand, while Gutiérrez-Lomelì *et al.*[42] examined strains producing 40-45 g/L of ethanol, we used physiological conditions leading to the accumulation of 4-6 g/L. Therefore, it could be speculated that a saturation limit could be reached when the strains are grown in the presence of a huge amount of glucose.

A minor difference is that Gutiérrez-Lomelì *et al.*[42] did not observe any improvement in the biomass production. Once more, it should be underlined that the transformed strains have been grown under very different conditions (rich-complex or defined-minimal media, respectively).

Guillaume *et al.*[51] have demonstrated that the pattern of fructose utilization during wine fermentation can be altered in yeasts harbouring a mutated *HXT3* allele. More in details the authors found that the glycolytic flux could be increased by the overexpression of a mutated version of the transporter gene. Data demonstrate that the Hxt3 hexose transporter plays a key role in determining the glucose/fructose utilization ratio during wine fermentation. All these findings are in line with the data shown in this paper as well as with the ones reported by Elbing *et al.*[15]. Following a very elegant approach, the authors built a series of strains having different rates of ethanol production, linearly correlating with the maximal specific glucose consumption rates attained during exponential growth on glucose. However, the same authors concluded that the hexose transporter has no or very low control over glycolytic flux in the wild type cells growing in the presence of high glucose concentrations.

Concluding, even if the metabolically engineered *S. cerevisiae *yeast strains are among the most prominent recombinant hosts usable for the industrial production of lactic acid [4,8,12,44], the overexpression of a hexose transporter has never been conceived to improve the productivity of this organic acid. In the recent past, aimed to improve the lactic acid production by metabolically engineered yeasts, we showed that the redirection of the pathway towards the lactate production can be strongly modulated by the genetic background of the host cell, by the source of the heterologous LDH enzyme, by improving its biochemical properties as well as by modulating (even if to very low extent) the export of lactate in the culture media [49]. In this article, we modulate the lactic acid productivity by improving the efficiency of the first step of the pathway - the glucose uptake - leading to the accumulation of lactic acid from glucose.

Finally, it should be underlined that a variety of organic acids draw more and more attention as new building block materials for the chemical industry [4]. If produced by environmentally benign fermentation strategies, they can provide a sound alternative to petroleum derived, and therefore limited, building block materials.

It can be anticipated that the production of these organic acids could be similarly improved by the overexpression of additional copies of one or more hexose transporters [43].

## Methods

### Yeast strains, transformation, media and cultivation

The *S. cerevisiae *strains used in this study derive from the following strains: GRF18U (*MATα*, *ura3; leu2-3,112; his3-11,15*; cir+) [45], CEN.PK strains 102-5B (*MATa, ura3-52, his3-11, leu2-3/112, TRP1, MAL2-8c, SUC2 *- Dr. P. Kötter, Institute of Microbiology, Johann Wolfgang Goethe-University, Frankfurt, Germany) [46], and CEN.PK m850 [50,52]. Yeast transformations were performed basically according to the LiAc/PEG/ss-DNA protocol [53]. The control strain is, for each background, the corresponding yeast strain transformed with the empty plasmid(s).

Independent transformants and the respective control strains (at least three for each transformation) were cultivated in shake flasks in minimal synthetic medium (1.34% or 2,68% [w/v] YNB medium [catalogue no. 919-15 Difco Laboratories, Detroit, Mich.] with 2% or 5% [w/v] glucose and 50 mg/L or 150 mg/L of appropriate amino acid(s), respectively).

All strains were grown in shake flasks at 30°C. For aerobic growth, flasks were agitated at 160 r.p.m. and the ratio of flask volume/medium was of 5/1. For microaerobic condition, flasks were sealed and the ratio of flask volume/medium was of 10/6.

Independent transformants derived from the strain CEN.PK m850 [LDH] were cultivated as previously described [52]. Briefly, growth kinetics were performed at 28°C in 250-mL quadruple baffled shake flasks in minimal medium containing 4.5 g/L CaCO_3_, 1.7 g/L YNB without amino acids and without (NH_4_)_2_SO_4_, 1 g/L urea, 5 g/L ethanol, and with glucose 9% (w/v) as a carbon source. Cell growth was monitored by measuring the optical density at 660 nm at regular time intervals.

### Gene amplification and plasmids construction

The *S. cerevisiae HXT*1 [47] and *HXT*7 [31] genes were PCR amplified using as a template the genomic DNA extracted from GRF18U strain by standard methods [54]. Pwo DNA polymerase (Roche catalogue no. 11 644 955 001) was used on a GeneAmp PCR System 9700 (PE Applied Biosystem, Inc.). Standard conditions used were 0.2 mM primers, 1.5 U of Pwo and 3 μL of genomic DNA. The program used for amplification of genes was as follows: after 5 min at 94°C, 30 cycles (each cycle consisting of 15 s at 94°C, 30 s at 57.5°C and 1 min 30 s at 72°C) were carried out, followed by 7 min at 72°C. Oligonucleotides pairs for *HXT*1 were as follows: HXT1_fw (5'-AAA ATC ATG AAT TCA ACT CCC GAT CTA-3') and HXT1_rev (5'-AGC TTG TTT AGT TTA TTT CCT GCTG AAA-3'). Because of the high sequence homology between the coding sequence of the *S. cerevisiae HXT*6 and *HXT*7 genes [47], the latter was amplified in two steps. In the first step the oligos named 5'HXT7 (5'-A AAA ATG TCA CAA GAC GCT GCT ATT GCA-3') and 3'HXT7exit (5'-ATA TAT TAA AAA CGT ATT TAC TTT TCA AGT-3') were used, the second designed on an external region in respect to the gene that resulted different from the corresponding region of the *HXT*6 gene. The single amplified band was secondarily used as a template for the two oligos 5'HXT7 and 3'HXT7 (5'-AGT GTC GAC AAA TAA TTT GGT GCT GAA CAT-3'), obtaining the sole open reading frame of the desired gene.

The amplified fragments were sub-cloned into the *Escherichia coli *vector pSTBlue obtaining, respectively, the plasmids pSTBlueHXT1 and pSTBlueHXT7. The inserts were sequenced and resulted identical to the deposited *S. cerevisiae *corresponding sequences (*HXT*1, GeneID: 856494 and *HXT*7, GeneID: 851943). These coding sequences were used for the construction of the integrative expression plasmids p022HXT1 and p022HXT7, respectively, utilizing the basic *S. cerevisiae *integrative expression plasmid pYX022 (R&D Systems, Inc., Wiesbaden, D). For the construction of the plasmid p022HXT1, the recipient vector was *Eco*RI cut, blunted and dephosphorylated, while the insert was *Mlu*I blunt/*Pml*I excised from the pSTBlueHXT1 plasmid. For the construction of the plasmid p022HXT7, the recipient and the pSTBlueHXT7 vectors were *Eco*RI cut. For the construction of the plasmid named p212LDH, the coding sequence of *L. plantarum *LDH was *Eco*RI excised from previously described pSTplLDH [49] and sub-cloned into the *S. cerevisiae *expression vector pYX212 (multicopy, *URA*3 auxotrophic marker R&D Systems, Inc., Wiesbaden, D) *Eco*RI opened and dephosphorylated. For the construction of the integrative plasmid p022KMX4, harboring an auxotrophic marker used only as a target gene and a dominant marker used for the selection of the transformants, the backbone of the plasmid pYX022 was used. pYX022 was *Kpn*I cut, blunt-ended and dephosphorylated and ligated with the Kan^R ^cassette derived from *Sph*I/*Sac*I blunt ending, from the plasmid pFA6KanMX4 [55]. p022KMX4 was *Eco*RI cut and dephosphorylated or *Eco*RI cut, blunt-ended and dephosphorylated and ligated with the *HXT*1 or *HXT7 *sequences cut as described above, resulting in the plasmids p022KMX4HXT1 or p022KMX4HXT7, respectively. A complete list of the transformed strains is given in Table 1.

DNA manipulation, transformation and cultivation of *E. coli *(Novablue, Novagen) were performed following standard protocols [54]. All the restriction and modification enzymes utilised are from NEB (New England Biolabs, UK) or from Roche Diagnostics.

### Metabolite determination

Residual glucose and ethanol/lactic acid produced were determined with enzymatic kits from Megazyme, the glucose assay kit (K-GLUHKR), the Ethanol kit (K-ETOH) and L-lactic acid kit (K-LATE), respectively, according to the manufacturer's instructions.

## Competing interests

The authors declare that they have no competing interests.

## Authors' contributions

GR participated in the design of the study and planned and carried out the experimental work including plasmid and strain construction, yeast cultivation and data analysis. MS participated in the design of the study and in data interpretation. DP and PB designed the whole study, performed data interpretation and drafted the manuscript. All authors read and approved the final manuscript.
